# Clinical Management of Migraine

**DOI:** 10.3390/jcm11175225

**Published:** 2022-09-04

**Authors:** Maria Albanese

**Affiliations:** 1 Regional Referral Headache Center, Neurology Unit, University Hospital Tor Vergata, 00133 Rome, Italy; maria.albanese@hotmail.it; 2 Department of Systems Medicine, University of Rome Tor Vergata, 00133 Rome, Italy

Migraine is one of the most frequent neurological and vascular disorders, with an estimated global prevalence of 14.4% [1]. It is characterized by recurrent episodes of unilateral pulsating pain associated with an altered sensitivity to light, sound, smell and touch.

People often experience severe autonomic manifestations such as nausea and vomiting, and in about 20% of cases, aura, consisting of reversible visual, sensory-motor or speech disturbances, can precede or accompany headache [1]. Headache persists for hours or even for days, causing negative effects in many aspects of life and daily activities during and between attacks [1].

Several trigger factors may increase migraine frequency over time, and a gradual worsening supervenes in up to a third of patients, leading to chronic and/or more refractory phenotypes [2]. Comorbidities are additional conditions that may co-occur with migraine (i.e., anxiety and depression, sleep disorders, cardiovascular diseases) and result in poorer health outcomes, more complex clinical management and increased socio-economic costs [2].

Notably, the Global Burden of Diseases (2019), Injuries, and Risk Factors Study ranked migraine as the first leading cause of disability in the working population (15–49 years) worldwide, with women affected nearly three times more often than men [3,4]. Therefore, an effective clinical management of this illness requires a combined approach to eliminate irregular lifestyle and specific precipitating factors, to control acute attacks, to contrast a progressive transformation and to remediate bothersome or disabling associated symptoms [1,2,3,4].

In recent decades, there has been a conceptual shift in understanding the pathology of migraine, away from a purely vascular model to the recognition that neuroinflammation and altered brain connectivity may have a key role in its pathogenesis [5]. This has led to the development of calcitonin gene-related peptide (CGRP)-targeted drugs—the major neuropeptide connected to migraine—that are dramatically changing some people’s lives and potentially moving the paradigm of management from a “symptomatic” to a “disease-modifying” point of view [5,6].

Currently, three humanized monoclonal antibodies (mAbs) directed against CGRP (Fremanezumab and Galcanezumab) or its receptor (Erenumab) are prescribed in clinical practice according to local guidelines for the preventive treatment of high-frequency and chronic migraine, with confirmed high efficacy and ability to reverse progression [7,8,9].

Growing evidence of favorable quick and long-lasting action, requiring less frequent administration, has made these medications preferred options over the standards of care, in terms of tolerability and adherence [6]. Many experts now do not exclude the possible use of these mAbs as first-line therapy for many patients with early disease, before severe disability is evident [10]. Nevertheless, other novel effective therapies acting as an agonist of the 5-HT1F receptor (Ditans) or as a CGRP antagonist (Gepants) are ready to emerge on the market for a better management of migraine crisis and/or its prevention, with expected good safety and tolerability profiles [11,12].

However, unsatisfactory and/or late response to anti-CGRP mAbs may occur in some migraine sufferers [13]. Add-on strategies such as neuromodulation, nerve blocks infiltrations and/or surgical decompression are available for treating more drug-resistant patients who show no improvement after medication intake [14].

Since CGRP is a potent vasodilator widely involved in many physiological and pathophysiological human processes [15], an important question is how long migraineurs can take these medications without experiencing any possible cardiovascular or other severe complications [16]. Moreover, another caveat to keep in mind is the administration of these drugs in potential “frail” categories, such as women who wish to become pregnant and patients with pre-existing major ischemic events and/or with ongoing immunomodulatory therapies [16,17]. In these scenarios, it may be also useful to consider alternative nonpharmacological modalities such as acupuncture, nutraceuticals, diet, relaxation or physical training and biofeedback in order to provide pain relief [18].

In conclusion, migraine is a global problem, and its prevalence is on the rise.

Although migraine disability and comorbidities usually guide the specialist to prefer one medication over another, a therapeutic failure may have psychologically detrimental effects. Given the heterogeneity conditions, the choice remains an individual alongside clinical guidance to consider benefits versus risks. Hence, the development of biomarkers able to predict the drug efficacy before its assumption is paramount for migraine management, decision making and personalized treatment success.
